# Structural Diversity and Biological Activities of Diterpenoids Derived from *Euphorbia fischeriana* Steud

**DOI:** 10.3390/molecules23040935

**Published:** 2018-04-18

**Authors:** Baiyu Jian, Hao Zhang, Jicheng Liu

**Affiliations:** 1 Graduate School of Heilongjiang University of Chinese Medicine, Heilongjiang University of Chinese Medicine, Harbin 150040, China; abcaaa1@126.com; 2 Research Institute of Medicine and Pharmacy, Qiqihar Medical University, Qiqihar 161006, China; zhang101hao@aliyun.com

**Keywords:** diterpenoids, *Euphorbia fischeriana* Steud, bioactivity

## Abstract

Diterpenoids are the focus of natural product drug discovery because of their great structural diversity and pronounced biological activities. *Euphorbia fischeriana* Steud is a Chinese traditional medicinal herb for curing edema, ascites, and cancer. This plant contains rich diterpenoids. Based on the carbon skeleton and substituents, it can be classified into thirteen subtypes: *ent*-abietane, daphnane, tigliane, ingenane, *ent*-atisane, *ent*-rosane, *ent*-kaurene, *ent*-kaurane, secotigliane, lathyrane, ent-pimarene, isopimarene and dimeric. In this paper, we reviewed the chemical structures and biological activities of 90 diterpenoids isolated from this medicinal herb. We hope that this work can serve as a reference for further research of these diterpenoids and lay the foundation for drug discovery.

## 1. Introduction

*Euphorbia fischeriana* Steud is a perennial herbaceous plant belonging to the family Euphorbiaceae, which is primarily distributed in northeastern mainland China [1]. The dried plant roots of *E. fischeriana* in traditional Chinese medicine, have been used as a remedy for the treatment of ailments, including edema, ascites and cancer [2,3,4]. The main components of *E. fischeriana* are diterpenoids, triterpenes, steroids, aromatic compounds and tannins [3]. Among them, diterpenoids are the primary bioactive constituents of this plant. Diterpenoids are a focus of natural product drug discovery because of their great structural diversity and pronounced biological activities [5]. In the last few years, a tigliane-type diterpenoid prostratin extracted from the roots of *E. fischeriana* has attracted great interest because of its unique ability to activate latent viral reservoirs and protect healthy cells from infection [5,6]. It is a protein kinase C activator that can reactivate HIV-1 latency and help to avoid the new infection of CD4+ Cells [5,6]. It brings new hope for the treatment of HIV. This result encouraged us to screen more bioactive diterpenoids from *E. fischeriana* for disease treatment.

In this review article, we focus on the structures and biological activities of 90 diterpenoids with different structures derived from *E. fischeriana*. We hope this work can serve as a reference for drug discovery.

## 2. Chemical Structure of Diterpenoids Derived from *E. fischeriana*

**Figure 1:**
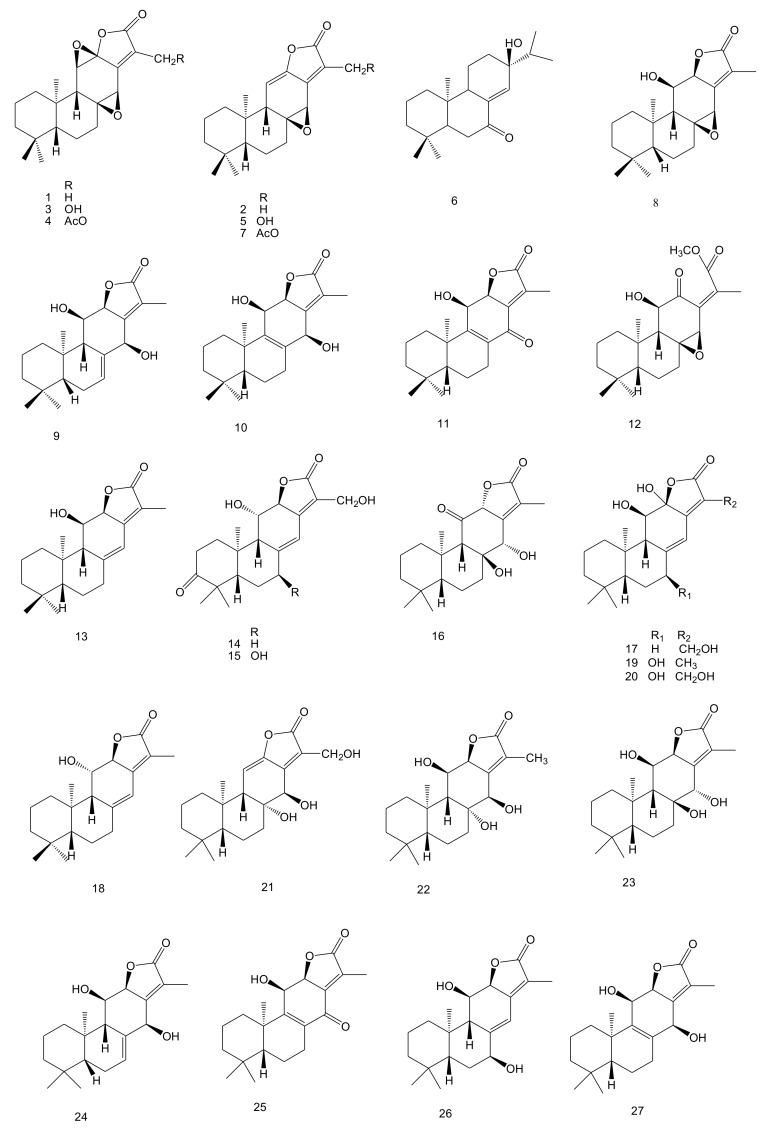

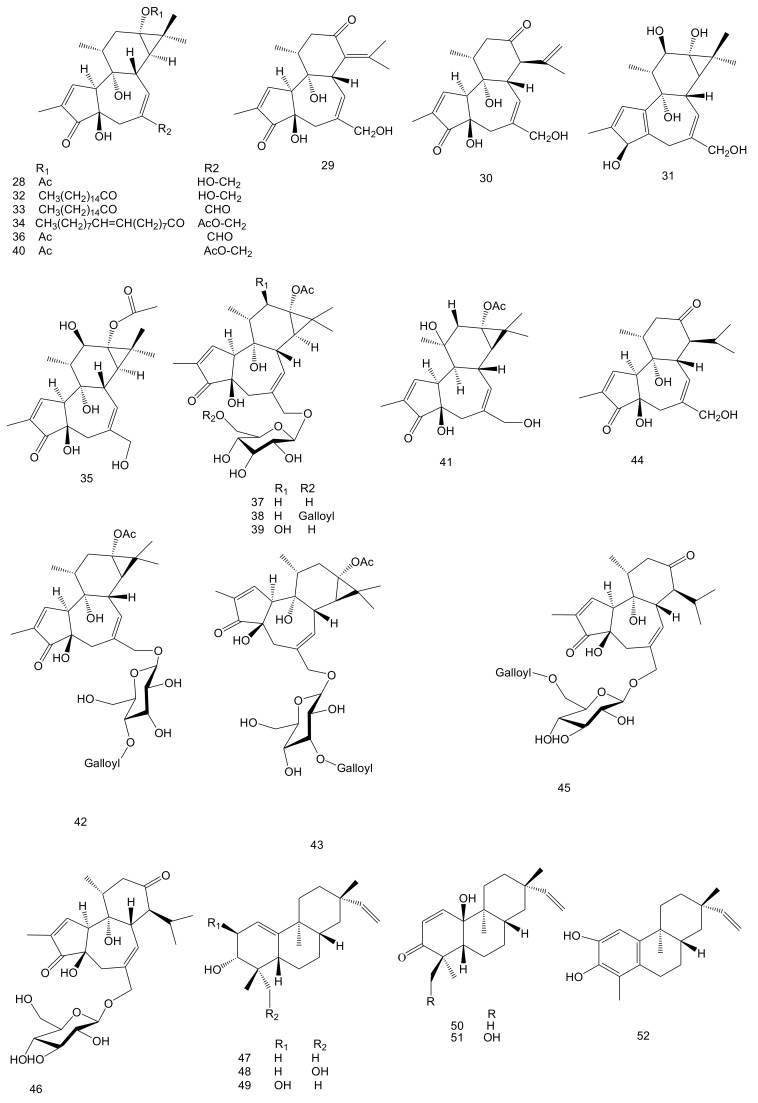

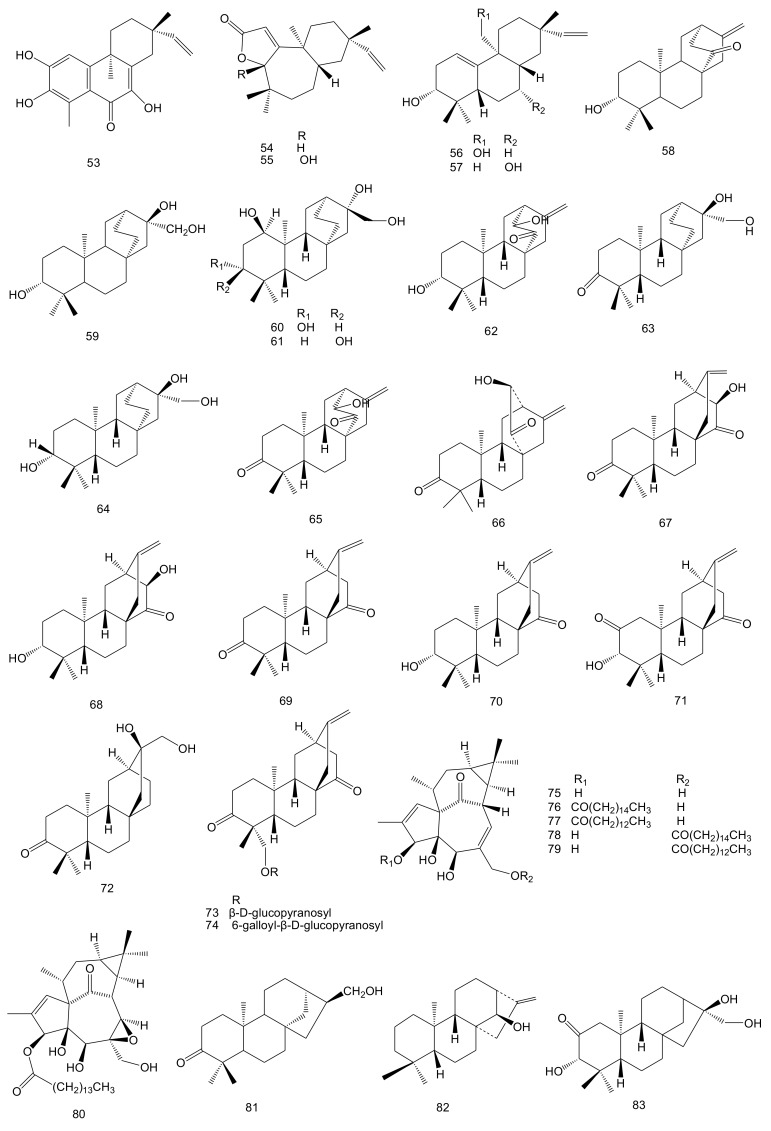

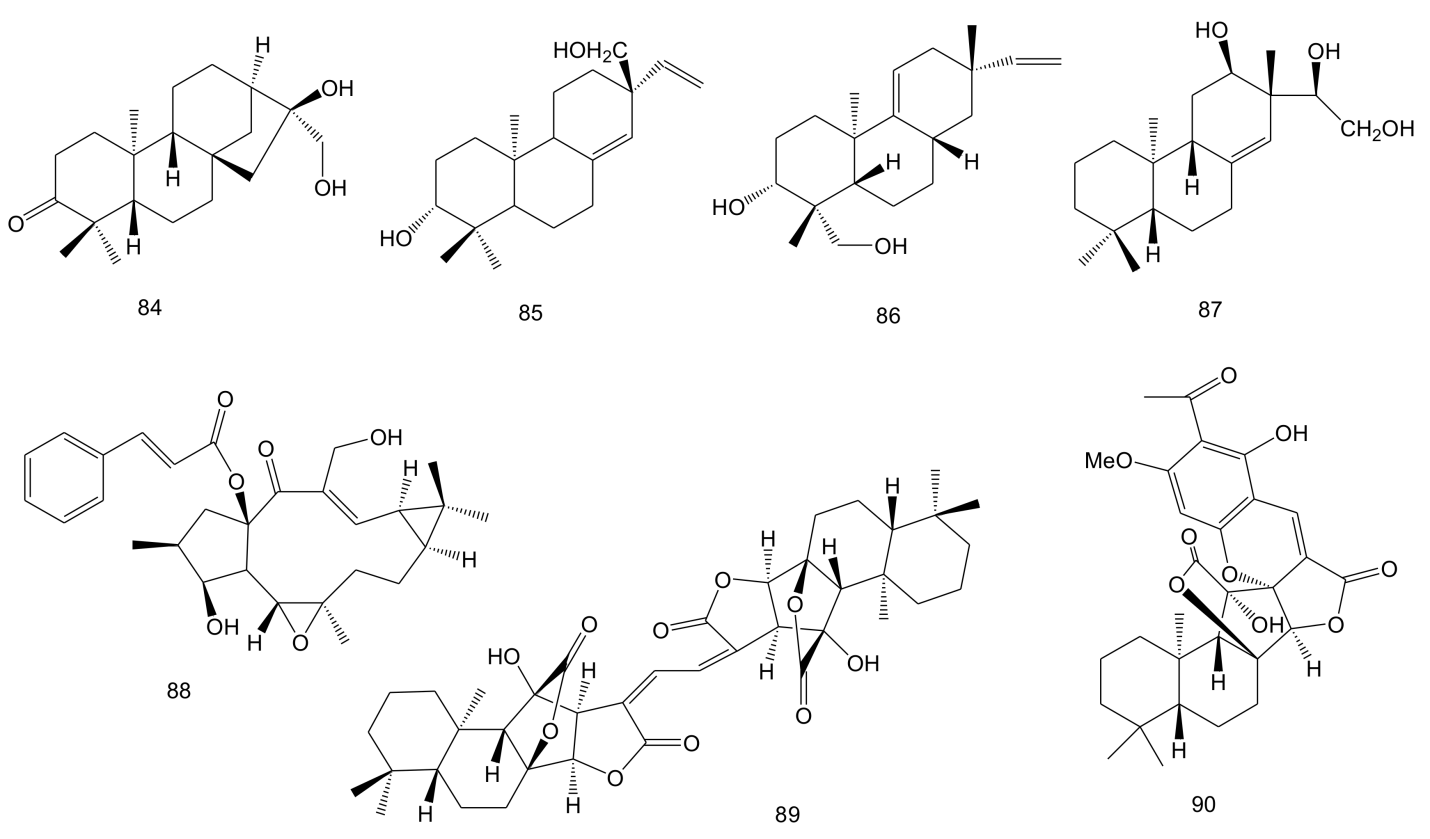
The chemical structures of diterpenoids derived from *E. fischeriana*.

Diterpenoids are the major components of *E. fischeriana*. According to the literature, almost 90 diterpenoids have been isolated from the roots of this plant since the 1970s. Types of diterpenoids include *ent*-abietane, daphnane, tigliane, ingenane, *ent*-atisane, *ent*-rosane, *ent*-kaurene, *ent*-kaurane, secotigliane lathyrane, *ent*-pimarene, isopimarene and dimeric. Their names, subtypes and corresponding references are compiled in Table 1. Their chemical structures (**1**–**90**) are shown in Figure 1.

## 3. Biological Activities of Diterpenoids

Diterpenoids isolated from *E. fischeriana* exert many different activities, including antitumor, anti-inflammatory, anti-HIV-1, feeding deterrent and others.

### 3.1. Antitumor Activity

Many investigations have been performed on the antitumor activity of diterpenoids from *E. fischeriana*. Compounds with different skeletal types (e.g., abietane, ingenane, tigliane and lathyrane) proved to have moderate or strong antiproliferative effects on different human cancer cell lines [5,15]. Jolkinolide B (**1**), a typical *ent*-abietane-type diterpenoid isolated from *E. fischeriana*, was found to induce time- and dose-dependent cytotoxicity in cells derived from liver [18], breast [19,20], gastric [18], cervical cancers [18], as well as human leukemic [21,22]. The anticancer effect of Jolkinolide B are associated with various kinds of mechanisms. Jolkinolide B is capable of inducing apoptosis in breast cancer cells through inhibition of the PI3K/Akt signaling pathway [19,20]. It can block cell cycles at G1 in human myeloid Leukemic cell K562 [22]. Moreover, it restrains the metastasis in breast cancer MDA-MB-231 cells through suppression of β_1_-integrin expression and phosphorylation of focal adhesion kinase (FAK) [23]. 17-Acetoxyjolkinolide B (**4**), has the potential to irreversibly inhibit IκB kinase and induce apoptosis of tumor cells. Nuclear factor-κB (NF-κB) plays an important role in tumor cell survival, growth, angiogenesis, and metastasis. 17-Acetoxyjolkinolide B is a novel type of anticancer drug candidate, as a NF-κB pathway inhibitor [24]. 17-Hydroxyjolkinolide B (**5**), a natural bioactive molecule, exerts its cytotoxicity against liver [18], breast 18], gastric [18], cervical [18], lung [18], ovarian [25], prostate [25], colorectal [25], cervix cancers [25]. Thorough examination proved that 17-hydroxyjolkinolide B can inhibit signal transducers and activators of transcription 3 signaling by covalently cross-linking Janus kinases and induce apoptosis of human cancer cells [25]. 12-deoxyphorbol 13-palmitate (**32**), a tigliane-type diterpenoid isolated from *E. fischerian* is another effective antineoplastic compound. 12-Deoxyphorbol 13-palmitate was found to mediate cell growth inhibition, G2-M cell cycle arrest and apoptosis in BGC823 cells [26]. It also reportedly has the ability to inhibit VEGF induced angiogenesis via suppression of VEGFR-2-signaling pathway [27]. Three ingenol diterpenoids including ingenol 3-palmitate (**76**), ingenol-3-myristinate (**77**), ingenol 6,7-epoxy-3-tetradecanoate (**80**) showed significant cytotoxicity against A549 with IC_50_ value of 3.35, 2.85, 2.88 μg/mL, respectively [2]. Two ent-atisane-type diterpenoids *ent*-1β,3β,16β,17-tetrahydroxyatisane (**60**), *ent*-1β,3α,16β,17-tetrahydroxyatisane (**61**) showed inhibitory effects against MCF-7 with IC_50_ levels of 23.21 and 15.42 mM [15]. Moreover, 13 diterpenoids, including jolkinolide B (**1**), euphorin E (**9**), euphorin H (**12**), yuexiandajisu E (**22**), ebractenoid C (**48**), ebractenoid F (**52**), euphorinC (**53**), *ent*-3β-hydroxyatis-16-ene-2,14-dione (**71**), 19-*O*-β-Dglucopyranosyl-*ent*-atis-16-ene-3,14-dione (**73**), ingenol-3-palmitate (**76**), ingenol-3-myristinate (**77**), ingenol-20-myristinate (**79**), and jolkinol A (**88**) showed inhibitory activity on mammosphere formation in human breast cancer MCF-7 cells at a final concentration of 10 μM, suggesting the potential of these bioactive diterpenoids for further investigation of the action targeting cancer stem cells [7,16]. The previous article provide more detailed descriptions about the anticancer mechanisms of these bioactive diterpenoids [28].

### 3.2. Anti-Inflammatory

Jolkinolide B (**1**) was reported to have a protective effect on LPS-induced ALI in mice [29]. It was revealed that jolkinolide B significantly inhibited LPS-induced histological alterations, lung edema, inflammatory cell infiltration, myeloperoxidase (MPO) activity [29]. At the molecular level, jolkinolide B reduced the production of TNF-α, IL-6 and IL-1β. Furthermore jolkinolide B was shown to inhibit LPS-induced the degradation of IκBα and phosphorylation of NF-κB p65 and MAPK [29]. 17-Hydroxy-jolkinolide B (**5**) was found to a potential anti-inflammatory drug candidate. It can inhibit LPS-induced the production of PGE 2, NO, IL-6, and TNF-α in RAW264 cells. 17-Hydroxy-jolkinolide B has the ability to reduce the expression of *COX-2*, *iNOS*, *IL-6*, and *TNF-α* gene through the suppression of MAPK phosphorylation and NF-κB activation. In addition, it induced the HO-1 expression [30]. Five diterpenoids including jolkinolide B (**1**), 11β-hydroxy-8,14-epoxy-*ent*-abieta-13(15)-en-16,12α-olide (**8**), yuexiandajisu D (**23**), ebractenoid F (**52**), jolkinol A (**88**) exhibited promising inhibitory effects on NO production in LPS-induced RAW 264.7 macrophages [8]. These compounds may be worthy of further investigation for the treatment of inflammatory diseases associated with enhanced production of NO [8]. Here, these studies are summarized in Table 2.

### 3.3. Anti-HIV-1

Prostratin (**28**), a tigliane-type diterpenoid, has been shown to be highly effective in inducing HIV-1 reactivation in latent reservoirs of infected Jurkat-LAT-GFP cells [5,31]. Five tigliane-type diterpenoids, including prostratin (**28**), fischeroside A (**37**), fischeroside B (**38**), fischeroside C (**39**), 12-deoxyphorbol-13,20-diacetate (**40**), were tested for cytotoxicities against C8166 cells. Compounds **37**–**39** showed weak activity in preventing the cytopathic effects of HIV-1 in C8166 cells [12]. Prostratin exerted the strongest anti-HIV-1 activity, with an EC_50_ of 0.00006 μM and a TI of 8500 [12]. 12-Deoxyphorbol-13,20-diacetate displayed anti-HIV-1 activity, with an EC_50_ of 0.003 μM and a TI of 366.67 [12]. This assay demonstrated that introducing an O-acetyl or glucopyranosyl moiety at C-20 of prostratin may dramatically reduce its anti-HIV-1 activity [12].

### 3.4. Feeding Deterrent

Feeding deterrent activities of 17-hydroxyjolkinolide A (**3**), 17-hydroxyjolkinolide B (**5**), jolkinolide B (**1**), 12-deoxyphorbol 13-(9*Z*)-octadecenoate 20-acetate (**34**) have been studied against two stored-product insects, *T. castaneum* and *S. zeamais* [4]. The experiment results are shown in Table 3. Jolkinolide B exhibited significant feeding deterrent activity against *S. zeamais* (EC_50_ = 342.1) and *T. castaneum* adults (EC_50_ = 361.4) [4].

### 3.5. Other Activities

Three diterpenoids 4β,9α,20-trihydroxy-13,15-secotiglia-1,6-diene-3,13-dione 20-*O*-β-d-[6-galloyl]glu-copyranoside (**45**), euphopiloside A (**46**), *ent*-8(14)-pimarene-12β,15*S*,16-triol (**87**) displayed moderate inhibitory effects against α-glucosidase [13]. These compounds could be applied to slow down the glucose level for diabetes [13]. 17-Hydroxyjolkinolide B (**3**) exhibited an inhibitory effect against mycobacterium smegmatis [1]. This means that it possesses a potential antituberculosis effect [1]. 17-Hydroxy-jolkinolide A (**5**) exerted an inhibitory effect on bone loss by preventing osteoclast formation and bone resorption [32]. 17-Hydroxy-jolkinolide A (**5**) treatment led to down-regulation of the expression of tartrate-resistant acid phosphatase (TRAP), cathepsin K (Cts K) and MMP-9 [32]. This compound may be useful as a therapeutic reagent for bone loss-associated diseases [32]. Here, we summarize these studies in Table 4.

## 4. Conclusions

Natural plants contain a variety of bioactive compounds. They are frequently used as drugs or lead compounds in drug development. Almost 70% of modern drugs have a natural product origin [5,33,34]. Therefore, searching for compounds with important biological activities from natural plants is of great significance. Diterpenoids are a constant focus of drug discovery because of their great structural diversity, resulting in various bioactivities. *E. fischeriana* is especially rich in diterpenoids [34]. In this paper, we have summarized 90 diterpenoids that have been isolated and identified from *E. fischeriana*, many of which are novel diterpenoids. Up to now, the majority of these diterpenoids have not been studied in terms of their biological activities. We hope to discover more potential drug leads in the future study. The mechanisms of bioactive ingredients from *E. fischeriana* need deeper research. Thoroughly understanding the targets for active compounds will help us to design effective new drugs. This review provides reference for further research of these diterpenoids and lays the foundation for drug discovery.

## Figures and Tables

**Table 1:** Emerging diterpenoids in *E. fischeriana*.

No.	Compound	Subtype	Ref.
1	jolkinolide B	*ent*-abietane	[3]
2	jolkinolide A	*ent*-abietane	[3]
3	17-hydroxyjolkinolide B	*ent*-abietane	[3]
4	17-acetoxyjolkinolide B	*ent*-abietane	[3]
5	17-hydroxyjolkinolide A	*ent*-abietane	[3]
6	13β-hydroxy-*ent*-abiet-8(14)-en-7-one	*ent*-abietane	[6]
7	17-acetoxyjolkinolide A	*ent*-abietane	[2]
8	11β-hydroxy-8,14-epoxy-*ent*-abieta-13(15)-en-16,12-olide	*ent*-abietane	[2]
9	euphorin E	*ent*-abietane	[7]
10	euphorin F	*ent*-abietane	[7]
11	euphorin G	*ent*-abietane	[7]
12	euphorin H	*ent*-abietane	[7]
13	*ent*-11α-hydroxy-abieta-8(14),13(15)-dien-16,12α-olide	*ent*-abietane	[7]
14	11α,17-dihydroxyhelioscopinolide E	*ent*-abietane	[1]
15	6β,11α,17-trihydroxyhelioscopinolide E	*ent*-abietane	[1]
16	11-oxo-ebracteolatanolide B	*ent*-abietane	[1]
17	7-deoxylangduin B.	*ent*-abietane	[1]
18	*ent*-11β-hydroxyabieta-8(14),13(15)-dien-16,12β-olide	*ent*-abietane	[2]
19	7β,11β,12β-trihydroxy-*ent*-abieta-8(14),13(15)-dien-16,12-olide	*ent*-abietane	[1]
20	langduin B	*ent*-abietane	[1]
21	(4*R*,4*aR*)-dihydroxy-3-hydroxymethyl-7,7,10*a*-trimethyl-2,4,4*a*,5,6,6*a*,7,8,9,10,10*a*,l0*b*-dodecahydrophenanthro[3,2-b]furan-2-one	*ent*-abietane	[1]
22	yuexiandajisu E	*ent*-abietane	[1]
23	yuexiandajisu D	*ent*-abietane	[8,9]
24	fischeriolide A	*ent*t-abietane	[8]
25	fischeriolide B	*ent*-abietane	[8]
26	fischeriolide C	*ent*t-abietane	[8]
27	fischeriolide D	*ent*-abietane	[8]
28	prostratin	tigliane	[3]
29	14-didehydrolangduin A	tigliane	[10]
30	langduin F	tigliane	[10]
31	3-hydroxyl-4-dehydro-10-dehydroxylphorbol	tigliane	[10]
32	12-deoxyphorbol 13-palmitate	tigliane	[3]
33	12-deoxyphorbaldehyde-13-hexadecacetate	tigliane	[3]
34	12-deoxyphorbol 13-(9*Z*)-octadecenoate 20-acetate	tigliane	[4,10]
35	13-*O*-acetyl-phorbol	tigliane	[11]
36	12-deoxyphorbaldehyde-13-acetate	tigliane	[3]
37	fischeroside A	tigliane	[12]
38	fischeroside B	tigliane	[12]
39	fischeroside C	tigliane	[12]
40	12-deoxyphorbol-13,20-diacetate	tigliane	[12]
41	9-deoxy-11β-hydroxyprostratin	tigliane	[1]
42	prostratin 20-*O*-(4′-galloyl)-β-d-glucopyranoside	tigliane	[1]
43	prostratin 20-*O*-(3′-galloyl)-β-d-glucopyranoside	tigliane	[1]
44	langduin A	daphnane	[3]
45	4β,9α,20-trihydroxy-13,15-secotiglia-1,6-diene-3,13-dione 20-*O*-β-d-[6-galloyl]glu-copyranoside	secotigliane	[13]
46	euphopiloside A	daphnane	[13]
47	*ent*-3β-hydroxy-rosa-1(10),15-diene	*ent*-rosane	[7]
48	ebractenoid C	*ent*-rosane	[7]
49	yuexiandajisu F	*ent*-rosane	[7]
50	euphorin A	*ent*-rosane	[7]
51	euphorin B	*ent*-rosane	[7]
52	ebractenoid F	*ent*-rosane	[7]
53	euphorin C	*ent*-rosane	[7]
54	fischeria A	*ent-*rosane	[7]
55	euphorin D	*ent*-rosane	[7]
56	3,20-dihydroxy-*ent*-1(10), 15-rosadiene	*ent*-rosane	[2]
57	3,7-dihydroxy-*ent*-1(10), 15-rosadiene	*ent*-rosane	[2]
58	*ent*-(3α,5β,8β,9α*,*10β,12β)-3-hydroxyatis-16-en-14-one	*ent*-atisane	[14]
59	*ent*-atisane-3α,16β,17-triol	*ent*-atisane	[14]
60	*ent*-1β,3β,16β,17-tetrahydroxyatisane	*ent*-atisane	[15]
61	*ent*-1β,3α,16β,17-tetrahydroxyatisane	*ent*-atisane	[15]
62	*ent-*3β,13S-dihydroxy-atis-16-en-14-one	*ent*-atisane	[15]
63	*ent*-16α,17-dihydroxyatisan-3-one	*ent*-atisane	[15]
64	*ent*-atisane-3β,16α,17-triol	*ent*-atisane	[15]
65	*ent*-13-hydroxyatis-16-ene-3,14-dione	*ent*-atisane	[2]
66	*ent*-13S-hydroxy-16-atisene-3,14-dione	*ent*-atisane	[12]
67	*ent*-13α-hydroxyatis-16-ene-3,14-dione	*ent*-atisane	[16]
68	*ent*-3β,13α-dihydroxyatis-16-ene-14-one	*ent*-atisane	[16]
69	*ent*-atis-16-ene-3,14-dione	*ent*-atisane	[16]
70	*ent*-3β-hydroxyatis-16-ene-14-one	*ent*-atisane	[16]
71	*ent*-3β-hydroxyatis-16-ene-2,14-dione	*ent*-atisane	[16]
72	*ent*-16α,17-dihydroxyatis-16-ene-3,14-dione	*ent*-atisane	[16]
73	19-*O*-β-Dglucopyranosyl-*ent*-atis-16-ene-3,14-dione	*ent*-atisane	[16]
74	19-*O*-(6-galloyl)-β-d-glucopyranosyl-*ent*-atis-16-ene-3,14-dione	*ent*-atisane	[16]
75	ingenol	ingenane	[7]
76	ingenol-3-palmitate	ingenane	[7]
77	ingenol-3-myristinate	ingenane	[7]
78	ingenol-20-palmitate	ingenane	[7]
79	ingenol-20-myristinate	ingenane	[7]
80	ingenol-6,7-epoxy-3-tetradecanoate	ingenane	[2]
81	*ent*-kaurane-3-oxo-17β-ol	*ent*-kaurane	[14]
82	*ent*-kaur-16-en-14-ol	*ent*-kaurene	[2]
83	3*S*,16*S*,17-trihydroxy-2-one-*ent*-kaurane	*ent*-kaurane	[15]
84	*ent*-16α,17-dihydroxy-kauran-3-one	*ent*-kaurane	[7]
85	3α,17-dihydroxy-*ent*-pimara-8(14),15-diene	*ent*-pimarene	[3]
86	isopimara-9(11),15-diene-3,19-diol	isopimarene	[2]
87	*ent*-8(14)-pimarene-12β,15S,16-triol	*ent*-pimarene	[13]
88	jolkinol A	lathyrane	[7]
89	langduin C	dimeric	[3,17]
90	langduin D	dimeric	[16]

**Table 2:** Summary of the anti-inflammatory activities of diterpenoids.

No.	Bioactive Ingredient	Model	Conclusions	Ref.
1	jolkinolide B	LPS-induced ALI mouse model	it has a protective effect on LPS-induced ALI in mice, the anti-inflammatory mechanism of JB may be attributed to its suppression of NF-κB and MAPK activation	[29]
		LPS-induced RAW 264.7 macrophages	it exhibited inhibitory effect on NO production (IC_50_ 4.9 μM)	[8]
5	17-hydroxyjolkinolide B	LPS stimulated RAW264 murine macrophages	it can inhibit inflammatory mediators but activate heme oxygenase-1 expression in LPS-stimulated murine macrophages	[30]
8	11β-hydroxy-8,14-epoxy-*ent*-abieta-13(15)-en-16,12α-olide	LPS-induced RAW 264.7 macrophages	it exhibited inhibitory effect on NO production (IC_50_ 12.6 μM)	[8]
23	yuexiandajisu D	LPS-induced RAW 264.7 macrophages	it exhibited inhibitory effect on NO production (IC_50_ 5.6 μM)	[8]
52	ebractenoid F	LPS-induced RAW 264.7 macrophages	it exhibited inhibitory effect on NO production (IC_50_ 7.4 μM)	[8]
88	jolkinol A	LPS-induced RAW 264.7 macrophages	it exhibited inhibitory effect on NO production (IC_50_ 9.4 μM)	[8]

**Table 3:** Feeding deterrent activities of diterpenoids from *Euphorbia fischeriana*.

No.	Compound	Insect Type	EC_50_ (ppm)	Ref.
1	jolkinolide B	*S. Zeamais* *T. castaneum*	342.1 361.4	[4]
3	17-hydroxyjolkinolide B	*S. Zeamais* *T. castaneum*	543.9 551.5	
5	17-hydroxyjolkinolide A	*S. Zeamais* *T. castaneum*	631.9 656.5	
35	12-deoxyphorbol 13-(9*Z*)-octadecenoate 20-acetate	*S. Zeamais* *T. castaneum*	884.3 1058.4	

**Table 4:** Other bioactive diterpenoids from *Euphorbia fischeriana*.

No.	Bioactive Ingredient	Pharmacological Activity	Ref.
3	17-hydroxyjolkinolide B	antituberculosis effect (it exhibited the inhibitory effect against mycobacterium smegmatis)	[1]
5	17-hydroxyjolkinolide A	anti-osteoporosis (it can prevent osteoclast formation and bone resorption)	[32]
45	4β,9α,20-trihydroxy-13,15-secotiglia-1,6-diene-3,13-dione 20-*O*-β-d-[6-galloyl]glu-copyranoside	anti-diabetic effect (it possesses the moderate inhibitory effects against α-glucosidase)	[13]
46	euphopiloside A	anti-diabetic effect (it possesses the moderate inhibitory effects against α-glucosidase)	[13]
87	*ent*-8(14)-pimarene-12β,15S,16-triol	anti-diabetic effect (it possesses the moderate inhibitory effects against α-glucosidase)	[13]
